# Bayesian Multivariate Modelling of Lone Star (*Amblyomma americanum*) Tick Life Stage Abundance and Temporal Trends to Inform Public Health Risk in Virginia

**DOI:** 10.3390/ijerph23050660

**Published:** 2026-05-15

**Authors:** Thabo Lephoto, Henry Mwambi, Oliver Bodhlyera, Holly Gaff

**Affiliations:** 1 School of Agriculture and Science, University of KwaZulu-Natal, Private Bag X01, Pietermaritzburg 3201, South Africa; mwambih@ukzn.ac.za (H.M.); bodhlyerao@ukzn.ac.za (O.B.); 2 Department of Biological Sciences, Old Dominion University, Norfolk, VA 23529, USA; hgaff@odu.edu

**Keywords:** vector ecology, spatio-temporal patterns, seasonal abundance, life stage dynamics

## Abstract

The increasing abundance of ticks poses a growing public health concern due to heightened human exposure to tick bites. The lone star tick (*Amblyomma americanum*: Ixodida: Ixodidae), a common human-biting species in the United States, has expanded its range in recent years. However, how its different life stages vary across time, habitats, and locations remains insufficiently understood. We analyzed tick abundance data collected in southeastern Virginia between 2009 and 2018, focusing on larval, nymphal, and adult life stages. A Bayesian multivariate modelling framework was used to examine seasonal patterns, habitat effects, spatial variation, and biological links between life stages. Two commonly used count models were compared to determine which best described the observed tick abundance patterns. Tick abundance showed strong and distinct seasonal patterns across life stages. Adult ticks were most abundant in late spring to early summer (May–June), nymphs peaked in early to mid-summer (June–July), and larvae peaked later in summer (August). Wooded habitats consistently supported higher tick abundance than grassy areas. Although both models captured these trends, the negative binomial model provided a more stable and biologically meaningful representation of tick dynamics. Several counties, including Chesapeake, York, Portsmouth, and Northampton, were identified as areas of elevated tick abundance, indicating increased tick bite exposure risk. This study highlights clear seasonal and habitat-specific windows of increased tick activity that are relevant for surveillance and control planning. By clarifying when and where different tick life stages are more abundant, the findings support targeted public health interventions aimed at reducing human exposure to tick bites in Virginia. The modelling approach is also applicable to other regions, including settings where ticks affect livestock health and food security.

## 1. Background

Ticks progress through three life stages, namely, larvae, nymph and adult, each requiring a blood meal from hosts such as mammals, birds, or reptiles, with habitat preferences for humid, vegetated areas like forests and grasslands [1]. Environmental factors, including temperature, humidity, and landscape changes, drive tick abundance and geographic range, with climate change and habitat fragmentation contributing to their increasing prevalence [1]. Increasing tick abundance and tick-borne pathogens constitute a growing threat to public health [2,3,4]. This tick abundance threatens public health by spreading diseases like *Ehrlichia chaffeensis* (causing human monocytic ehrlichiosis), Heartland virus, *Rickettsia amblyommatis*, and the agent of alpha-gal syndrome, a meat allergy triggered by tick bites [5].

A recent study in Kentucky reported detection of *Ehrlichia chaffeensis* in 32 counties out of the 77 sampled counties in 2019 through 2020 [5]. A 2011 Centers for Disease Control and Prevention (CDC) report indicated that there were 863 human cases of ehrlichiosis in the United States. An increase has been observed over the seven-year period, with human cases steadily increasing to 1832, with 1799 reported to be a result of *E. chaffeensis* infection [6,7].

Due to the effects of climate change and transportation by hosts, tick ranges remain ever-changing. In recent years, the *Amblyomma americanum* species has expanded its range into northeastern and midwestern United States and northward into Canada [8]. In eastern United States, the lone star tick (*Amblyomma americanum*) is one of the five major tick species transmitting pathogens of both human and veterinary significance. In Virginia, *A. americanum* is among the most frequently encountered ticks by humans and a common human-biting species [9]. *Amblyomma americanum* ticks have long been identified as nuisance biters because of their aggressive questing behaviour [10,11,12].

Despite its prominence as a nuisance biter and vector of human pathogens, efforts to define the species’ variations within counties remain limited [13]. Therefore, one of the strategies to help reduce tick-borne diseases is to conduct an in-depth analysis of life stage abundance patterns and identify factors contributing to abundance. As a result, our research focused on modelling *A. americanum* tick life-stage abundance collected over time from three different habitat types, namely, edges, woods and grass, across 13 random sample sites. The objective of this study was to identify abundance levels of *A. americanum* variations within sample sites from eight counties in the state of Virginia, United States. We also investigate the influence of habitat type, lagged stages and temporal predictors on tick abundance in Virginia.

Tick abundance is shaped not only by abiotic conditions but also by host availability. Larval and nymphal stages commonly feed on small mammals and birds, whereas adult *A. americanum* rely heavily on larger hosts, particularly white-tailed deer. Variations in host density, movement patterns, and habitat use can therefore influence life-stage-specific abundance and spatial heterogeneity. These ecological dependencies suggest that life stages are not independent processes but are biologically linked through survival and host-mediated progression.

Despite the ecological importance of stage-specific dynamics, many previous studies have relied on univariate models that examine each life stage separately. Such approaches do not explicitly account for interdependencies across stages or temporal structure inherent in tick development. In addition, ecological count data are often characterized by substantial variability and frequent zero observations, requiring flexible statistical frameworks capable of capturing overdispersion and spatio-temporal dependence.

Our study addresses this gap by applying a multivariate framework that incorporates life-stage lag effects and spatio-temporal random components. Similar multivariate count time-series frameworks have been applied in other domains, including finance [14], transportation [15,16], and therapeutic categories [17]. These models have largely been used for non-ecological applications or single-outcome extensions. We extend this framework by explicitly modelling lagged dependencies across tick life stages within a spatio-temporal ecological context. To our knowledge, this represents the first application of such a multivariate dynamic count framework to multistage tick abundance data at this spatial and temporal scale.

## 2. Methods

### 2.1. Data Description

This study used tick abundance data collected and prepared by researchers from the Department of Biological Sciences at Old Dominion University, Virginia, United States. Ticks were collected at least once a month from May 2009 through December 2018 across southeastern Virginia, using standard flagging techniques along established transects [18]. Each site consisted of one to four transects, depending on the size and habitat heterogeneity, with individual transects ranging from approximately 100 to 3000 m in length. Collected specimens were identified to species and life stage (larvae, nymphs, and adults) following the taxonomy of [19]. Sampling was conducted across three habitat types, edge, wooded, and grassy, to capture variation in tick abundance associated with land cover.

The original dataset included observations from 12 sampled locations across eight counties: York, Chesapeake, Norfolk, Hampton, Isle of Wight, Virginia Beach, Northampton, and Portsmouth. These locations included Jacobson Track (JC) and Stephens Tract (ST) in Chesapeake; Langley (LA) in Hampton; Blackwater Ecological Preserve (BW) in Isle of Wight; Weyanoke (WS) in Norfolk; Kiptopeke State Park (KP) in Northampton; Hoffler Creek Wildlife Preserve (HC) and Paradise Creek Nature Park (PC) in Portsmouth; Back Bay National Wildlife Refuge (BB) and Oceana/Dam Neck (OD) in Virginia Beach; and Naval Supply Center (CA) and Newport News Park (NN) in York.

To improve clarity, detailed geographic information for each sampling site, including county, site code, and geographic coordinates, is provided in Supplementary Table S1. Note that no altitude information was available in the original dataset, and as a result, we could not incorporate the covariate in the statistical analysis. However, potential environmental differences between sites were accounted for through location-specific random effects in the modelling framework.

The temporal dynamics of tick life stages are illustrated in Figure S1 (Supplementary Materials), which presents the monthly time series of larvae, nymph, and adult counts over the study period. The figure reveals pronounced seasonal peaks and clear temporal variation across life stages, with larvae exhibiting the highest variability and peak magnitudes. These patterns provide preliminary evidence of strong seasonality and potential temporal dependence between life stages. Figures S2 and S3 (Supplementary Materials) depict tick data collection sites in Virginia. Figure S2 shows a full map of Virginia with counties where data were collected highlighted and labelled with county names, while Figure S3 shows a zoomed-in portion of the southeastern Virginia where map shown in Figure S2. Table S1 shows all county boundaries and names, codes, and corresponding county names.

The full breakdown of tick counts across all sites and habitat types is shown in Table S2 of Supplementary Materials. From the table, it is evident that several locations had very few sampling events or zero sampling events in one or more habitat type. To improve data quality and avoid model identifiability issues related to sparse counts, we restricted our analysis to a subset of six locations that had consistent and sufficient sampling coverage of at least 104 total visits across habitat types and locations. The locations BB, BW, CA, KP, LA and OD provided adequate representation of at least two habitat types per site and sufficient variability in tick counts to support stable multivariate modelling (Table S2).

Table S3 in the Supplementary Materials summarizes the number of sampling events across the six retained locations and three habitat types. A value of zero indicates that no sampling visits were conducted in the habitat type at the corresponding location, and therefore, no tick life stage data were collected. For example, wooded habitat types were not sampled at BB, and edge habitat types were not sampled at BW.

### 2.2. Exploratory Data Analysis

Table 1 summarizes the basic characteristics of tick counts for larvae, nymphs, and adults and helps describe how the data are distributed. The table shows that tick counts are highly uneven, with many sampling occasions recording no ticks at all, while a few occasions record very large numbers. For example, although the average number of larvae was relatively high, most samples contained no larvae. This pattern indicates that tick counts vary much more than would be expected if ticks were evenly distributed across space and time. Similar patterns were observed for nymphs and adults, where a large proportion of samples also recorded zero ticks. These features suggest that ticks are not consistently present at all sites or times but instead occur sporadically, with occasional high-abundance events. Because the Poisson model assumes that counts fluctuate evenly around the average, it cannot adequately represent data with this level of variability and frequent absences. For this reason, we considered more flexible models that are better suited to ecological count data. The negative binomial model allows for greater variability in counts, while the zero-inflated negative binomial model additionally accounts for the large number of zero observations. Based on the data patterns observed in Table 1 and standard diagnostic checks, Poisson-based models were not appropriate and were therefore excluded from further analysis. A detailed exposition of count data models can be found in [20,21,22,23], among others.

**Figure 1:**
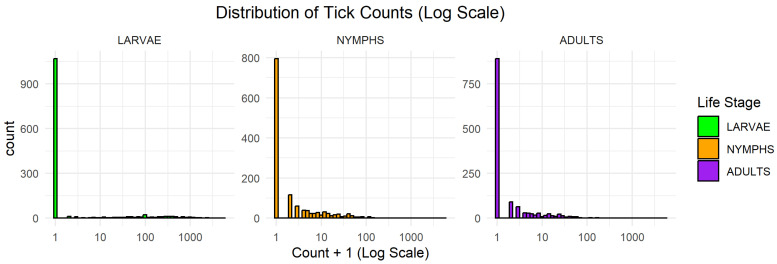
Distribution of observed tick counts for larvae, nymphs, and adults across all sampling sites (log[count + 1] scale). The strong right-skew and high frequency of zero observations highlight substantial variability and support the use of flexible count models.

Figure 1 shows the log-transformed tick counts (log[count + 1]) to improve the visualization of highly skewed distributions and extreme values. The log transformation was chosen over other options to handle the severe positive skew and multiple orders of magnitude in the tick counts. This approach effectively compressed extreme outliers and expanded the lower range, avoiding the ‘clumping’ of data near zero and ensuring more interpretable plots. All life stages exhibit strong right-skewness and a high frequency of zeros, particularly larvae, confirming substantial overdispersion. This visual pattern aligns with the descriptive statistics in Table 1 and confirms the presence of overdispersion and excess zeros in the data.

**Figure 2:**
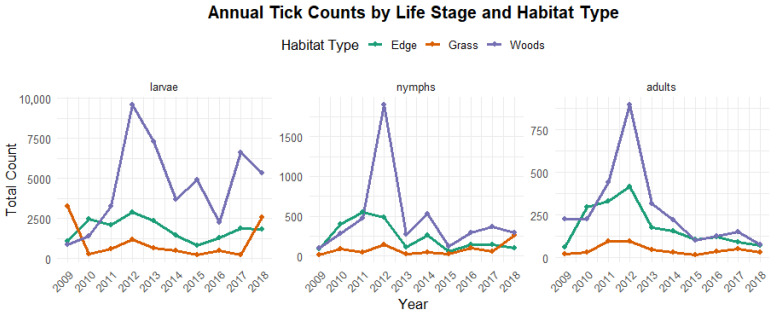
Annual tick abundance trends by habitat type (2009–2018). Wooded habitats consistently show higher tick counts across life stages compared to edge and grassy habitats, indicating greater environmental suitability.

**Figure 3:**
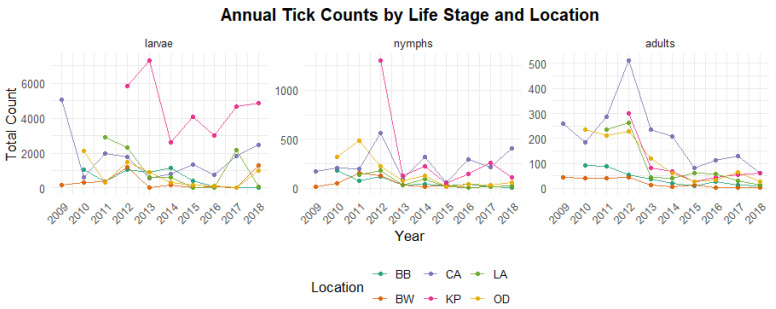
Annual tick abundance trends by sampling location (2009–2018). Certain locations (e.g., KP and CA) exhibit consistently higher tick counts, suggesting localized hotspots of tick activity.

Figure 2 shows the distribution of tick stages across habitat types. It is evident that wooded areas consistently recorded highest counts for all tick life stages, followed by edge habitat types, while the lowest counts were observed in grassy areas. In contrast, Figure 3 presents the annual distribution of tick counts by life stage and location. The highest numbers of larvae and nymphs were recorded at Kiptopeke State Park (KP), while adult ticks were most abundant at the Naval Supply Center (CA). In comparison, other locations exhibited relatively similar and lower tick counts across stages. It is also worth noting that CA and Oceana/Dam Neck (OD) recorded notably high adult tick counts between 2009 and 2013, whereas other locations had substantially lower adult tick activity during this period.

### 2.3. Statistical Modelling

For practical situations where responses arise as a vector of counts that vary across different observational sample sites/locations, univariate regression models for each of the components in the response vector cannot account for the association or dependence among the components of the response vector. The dependence may be due to omitted variables which simultaneously affect the response vector, [24,25], and therefore a multivariate or joint modelling approach is needed. However, in the case of tick stages, which define the components of the response vector, dependence between the stages is expected, because individuals in stage j+1 depend on the survival of individuals from stage j. We modelled the tick counts of *A. americanum* for each life stage j (where j=1 for larvae, j=2 for nymphs, and j=3 for adults) at location i and time t, employing a multivariate framework with the integrated Nested Laplace Approximation (INLA) by [26]. The response variable $Y_{j,it}$ represents the count of ticks for stage j at location i and time t, aggregated monthly from 2009 to 2018 across a refined dataset of 1307 observations. The dataset was derived from an original total of 4554 individual observations, which were first aggregated to 1758 records by habitat type, month, year and location, subsequently narrowed to well-sampled locations (BB, BW, CA, KP, LA, OD) to enhance model identifiability, estimability and reliability. Full mathematical formulations, prior specifications, and implementation details are provided in Supplementary Materials.

### 2.4. Model Framework

**Figure 4:**
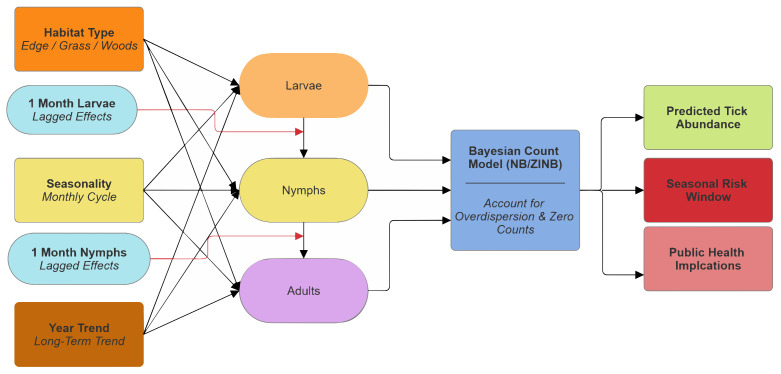
Conceptual diagram of the modelling framework. Tick abundance at each life stage is influenced by habitat type, seasonal effects, long-term temporal trends, spatial variation, and biologically linked lag effects indicated by red arrows between successive stages.

We modelled monthly counts of *A. americanum* larvae, nymphs, and adults using a Bayesian multivariate regression framework to examine how tick abundance varies across time, habitat type, and sampling location in Virginia. Because the three life stages are biologically linked through the tick life cycle, they were analyzed jointly rather than as independent outcomes, allowing dependencies between stages to be explicitly represented. Habitat type, seasonal variation, and long-term temporal trends influence the abundance of *A. americanum* at each life stage (larvae, nymphs, and adults). Biological progression between life stages is represented through lagged effects linking larvae to nymphs and nymphs to adults. Tick counts are analyzed jointly using Bayesian count models (negative binomial and zero-inflated negative binomial), which account for overdispersion and excess zero counts. Model outputs are used to estimate tick abundance, identify seasonal risk windows, and inform public health implications (Figure 4).

Tick count data commonly exhibit substantial variability and a high frequency of zero observations. To accommodate these features, we considered two flexible count models: the negative binomial (NB) model and the zero-inflated negative binomial (ZINB) model. The NB model allows the variance of tick counts to exceed the mean, which is typical of ecological population data characterized by aggregation and environmental heterogeneity. The ZINB model extends this formulation by allowing for additional zero counts beyond those expected under the NB process.

In ecological surveys, zero counts may arise either because ticks are truly absent from a site or because they are present but not detected during sampling. The ZINB model explicitly represents this distinction by including a separate component for excess zeros, whereas the NB model assumes that all zeros arise from the same ecological process governing non-zero counts.

Covariates included habitat type, seasonal effects, and biologically motivated lagged effects linking successive life stages. Tick abundance is influenced by climatic conditions such as temperature and rainfall, which regulate development rates, survival, and questing behaviour. However, high-resolution climate data corresponding to the irregular sampling schedule were not consistently available for all locations and months in the study period. Consequently, rather than including explicit meteorological covariates, seasonal patterns were captured using sinusoidal (cyclic) functions to represent recurring annual variation in tick activity. Lagged effects were included to represent biological progression between stages, whereby nymphal abundance depends on larval abundance in the preceding month, and adult abundance depends on preceding nymphal abundance. Habitat type was included to account for differences in environmental suitability across sampling locations.

To capture longer-term temporal variation shared across all life stages, a smooth year effect was included, allowing tick abundance to increase or decrease gradually over time. Additional random effects were included to account for unobserved heterogeneity across locations and life stages, reflecting differences in local habitat conditions and host availability.

To assess the robustness of our results to prior assumptions, we fitted the model under three alternative prior configurations. These prior sets differed only in the strength of the assumptions placed on the temporal and spatial random effects. One configuration represented the default weakly informative setting, while the second imposed stronger smoothing on temporal and spatial variation, and the third allowed greater flexibility by relaxing these constraints. This sensitivity analysis allowed us to evaluate whether biological interpretations and model selection were driven by prior assumptions or by the observed data. Model performance was evaluated using information criteria that balance goodness of fit with model complexity, alongside visual diagnostics comparing observed and fitted values and assessing predictive calibration. Based on these diagnostics, the NB model was selected as the primary model due to its stability and its ability to capture biologically realistic relationships between tick life stages without overfitting zero counts. All analyses were conducted using R version 4.3.2 [27] within the RStudio environment version 2026.1.0.392 [28].

## 3. Results

We evaluated two models for tick population data: a negative binomial (NB) and a zero-inflated negative binomial (ZINB), to address overdispersion, driven by habitat variability and sampling differences, and frequent zero counts when ticks are absent. Model performance was compared using the Deviance Information Criterion (DIC) and Watanabe–Akaike Information Criterion (WAIC) (Table 2). Although the ZINB had marginally lower DIC and WAIC values, we argue that the NB offers a superior fit based on ecological interpretability, parsimony, and robustness.

Specifically, NB estimated positive correlations between life stages, which aligned with expected biological dependencies. In contrast, ZINB produced a negative correlation between larvae and adults, likely an artefact of overcompensating for zero inflation (Supplementary Materials). Lee et al. [29] argue that it is important to consider other data characteristics when choosing a model for count data. Since zeros in our data are covariate-driven, and the model estimated 60.5% structural zeros for larvae, but only 0.010 and 0.012 for nymphs and adults, we argue that this might be an indication of potential overfitting to larvae zeros at the expense of other stages. Figure S4 shows that the proportion of zero counts varies systematically across habitat types for all tick life stages. Larvae exhibit the highest frequency of zeros, particularly in grass habitats, while nymphs and adults display substantially lower and habitat-dependent zero proportions. This heterogeneity across habitats is inconsistent with a common structural zero-generating mechanism and instead suggests that zero counts are driven by environmental covariates associated with habitat characteristics. Figure S5 demonstrates pronounced seasonal variation in the proportion of zero counts across all life stages. Zero frequencies are highest during winter months and decline substantially during late spring and summer, coinciding with periods of increased tick activity. These strong temporal patterns provide direct empirical evidence that zeros are seasonally driven and therefore attributable to covariates such as month and climatic conditions rather than arising from a separate structural zero process. Figure S6 further reinforces the covariate-driven nature of zero counts by illustrating the joint effects of habitat and season. Distinct clusters of high zero proportions are observed in specific habitat–month combinations, particularly for larvae, while periods of reduced zero frequency align with peak seasonal activity. The presence of these structured patterns across environmental covariates is inconsistent with random or structural zero inflation and supports modelling zero counts through covariates and overdispersion within the NB framework. Taken together, Figures S4–S6 and the exploratory data analysis demonstrate that zero counts in the tick abundance data are systematically associated with habitat type and seasonality rather than arising from a separate structural zero-generating process. Zeros occur predominantly under specific environmental and temporal conditions, with strong life-stage-specific patterns that align with known tick phenology. This empirical evidence supports modelling zeros through covariate effects and overdispersion, providing a principled justification for the use of the NB model over a zero-inflated alternative.

### 3.1. Model Diagnostics

**Figure 5:**
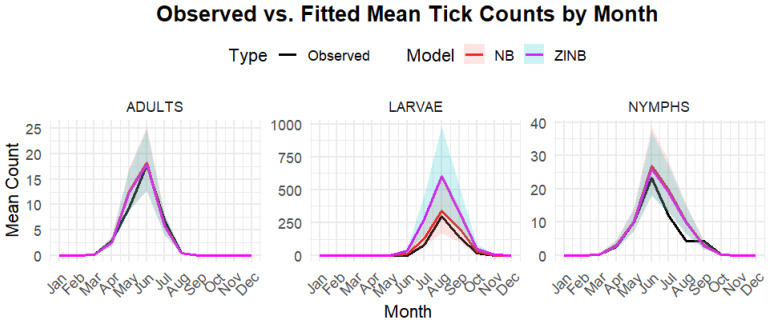
Monthly mean observed tick counts (black) compared with fitted values from the negative binomial (NB) model (red) and the zero-inflated negative binomial (ZINB) model (purple). The NB model closely reproduces seasonal peaks without overestimating extreme larval counts.

Posterior predictive checks and diagnostic plots were used to evaluate whether the competing models adequately represented the observed patterns of tick abundance. Both the negative binomial (NB) and zero-inflated negative binomial (ZINB) models reproduced the broad seasonal trends observed across tick life stages; however, the NB model more consistently matched the magnitude and timing of observed peaks without generating exaggerated high counts; see Supplementary Materials, Figure S7. Residual diagnostics indicated that the ZINB model showed greater instability at higher abundance levels, particularly for larvae, suggesting overfitting of infrequent extreme observations. In addition, probability integral transform (PIT) diagnostics for the ZINB model revealed systematic departures from uniformity, indicating model miscalibration and a tendency to underestimate observed counts during periods of high tick activity. Taken together, these results suggest that excess zeros in the data are sufficiently explained by ecological covariates and overdispersion, rather than by a separate structural zero-generating process. For reasons of ecological interpretability, stability, and parsimony, the NB model was therefore selected for inference and reporting. Figure 5 and Figures S7–S9 in Supplementary Materials show that while both models produced similar fits for adult and nymph tick stages, the ZINB model (purple curve) deviates more substantially from the observed values (black curve) compared to the NB model for the larval stage. Thus, the discrepancy indicated that the NB model provided a better fit to the data than the ZINB.

### 3.2. Posterior Parameter Estimates

The results presented in Table 3 stem from a negative binomial (NB) model fitted to multivariate tick count data for *A. americanum* across its larval, nymphal, and adult stages, incorporating spatial random effects. The analysis was carried out using the INLA framework and evaluates the influence of sinusoidal terms to capture monthly variation, habitat types and lagged larvae and nymph abundance to assess the influence of larvae on nymphs and nymphs on adult tick counts, while accounting for overdispersion.

Three distinct prior sets were employed to assess the robustness of parameter estimates. These prior configurations differed in the strength of assumptions imposed on the temporal (year) and spatial random effects. The first set represented the default weakly informative prior specification. The second imposed stronger smoothing on temporal and spatial variation, while the third allowed greater flexibility by relaxing these smoothing constraints. By comparing results across these configurations, we evaluated whether model stability and ecological interpretations are sensitive to prior assumptions or driven primarily by the observed data.

Model fit was assessed via the Deviance Information Criterion (DIC) and Watanabe–Akaike Information Criterion (WAIC). The interpretation integrates insights from exploratory data analysis (EDA) to contextualize the findings within the ecological and public health framework of the study.

Table 3 presents the posterior summaries of fixed effects, model hyperparameters, and model diagnostics across three prior specifications (Set 1–3). The table evaluates sensitivity to prior assumptions while providing insights into covariate effects, model structure, and the robustness of biological inferences drawn from the data.

The seasonal (harmonic) covariates showed strong and consistent associations with tick abundance across life stages. The coefficients for the sine and cosine larval-specific terms were significant, with $\beta_{sine_{L}}\approx -5.29,\;CI=\;[-5.74,-4.84]$ and $\beta_{cosine_{L}}\approx -2.49,\;CI\approx \;[-2.89,-2.08]$, indicating strong seasonal cycles for larvae. Similar patterns were observed for nymphs ($\beta_{sine_{N}}\approx -0.43,CI\approx [-0.62,-0.24]$, $\beta_{cosine_{N}}\approx -3.62,CI\approx [-3.89,-3.36]$) and adults ($\beta_{sine_{A}}\approx 1.15,\;CI\approx [0.95,\;1.36]$, $\beta_{cosine_{A}}\approx -5.09,\;CI\approx [-5.48,\;-4.70]$), reflecting distinct seasonal peaks and troughs in abundance.

The coefficients $\beta_{Grass}$ and $\beta_{Woods}$ represent the log-rate change in tick abundance relative to the edge reference category. Across all prior sets, the posterior means for $\beta_{Grass}$ is consistently negative ($\beta_{Grass}\approx -1.30,\;CI\approx \;[-1.48,-1.11]$) with narrow credible intervals, suggesting that grassy habitat types are associated with significantly lower tick abundance relative to the edge habitat type reference category. This aligns with the preliminary results in the exploratory data analysis (EDA) section, which showed lower counts of ticks across all life stages in grassy habitat types. Conversely, posterior means for wood habitat types were consistently positive and significant ($\beta_{Woods}\approx 0.50$, CI≈ [0.32, 0.67]) across priors, indicating elevated counts in wooded areas compared to the edge baseline. These findings align with ecological expectations given the microclimatic suitability of wooded habitat types for ticks and confirms the results in the EDA. Also, the results suggest that woods act as a favourable microhabitat, while grass areas are relatively less suitable.

The location- and habitat-type-specific lagged effects of larvae and nymphs on the current life stage counts were both positive and consistent across all prior sets ($\beta_{\log{(L}_{t-1})}\approx 0.21,CI\;\approx \;[0.15,\;0.28]$; $\beta_{\log{(N}_{t-1})}\approx 0.11,\;CI\;\approx \;[0.02,\;0.20]$). This supports the biological plausibility of stage progression, where higher past abundances predict higher current counts, particularly across adjacent life stages. Similar patterns are observed for other lagged covariates, supporting the EDA findings that stage-wise peaks are not always tightly aligned month-to-month.

The precision for the year random effect ($\tau_{year}$) was consistent in prior sets 1 and 3 ($\tau_{year}\approx 6.91$ and 6.96 with $CIs\approx \left[5.20,\;8.92\right]$ and [4.42, 10.38], respectively), indicating moderate interannual variability. In contrast, prior set 2 produced a substantially inflated posterior mean ($\tau_{year}\approx 20.28$) accompanied by extremely wide uncertainty (CI≈ [0.84, 110.49]), with excessive posterior standard deviation. This wide credible interval indicates an unstable estimate under this prior specification, reflecting strong prior sensitivity and reduced stability of the parameter for the year effect. Spatial precision also remained stable, although with wider uncertainty for set 2. The larvae and nymph stages exhibited significant and moderate spatial variation ($\;\tau_{loc3d\_L\;}\approx 1.60\;\tau_{loc3d_{N}}$ and $1.80;\;CIs\approx \left[1.26,2.00\right]$ and [1.32, 2.26]), while adult ticks showed spatial variability ($\tau_{loc3d_{A}}\approx 0.26;\;CI\approx \;[0.21,\;0.33]$), potentially reflecting their wider movement or dependence on localized hosts or environmental features. The model showed that correlations among life stages were consistently positive, especially between nymphs and adults ($\rho_{N:A}\approx 0.78;\;CI\approx \;[0.66,\;0.87]$), indicating shared spatio-temporal drivers or biological linkage between stages. The larva–nymph correlation ($\rho_{L:N}\approx 0.66;\;CI\approx \;[0.55,\;0.76]$) and larva–adult correlation ($\rho_{L:A}\approx 0.46;\;CI\approx \;[0.19,\;0.69]$) were moderate, suggesting that while life stages are connected, they also retain stage-specific variation.

**Figure 6:**
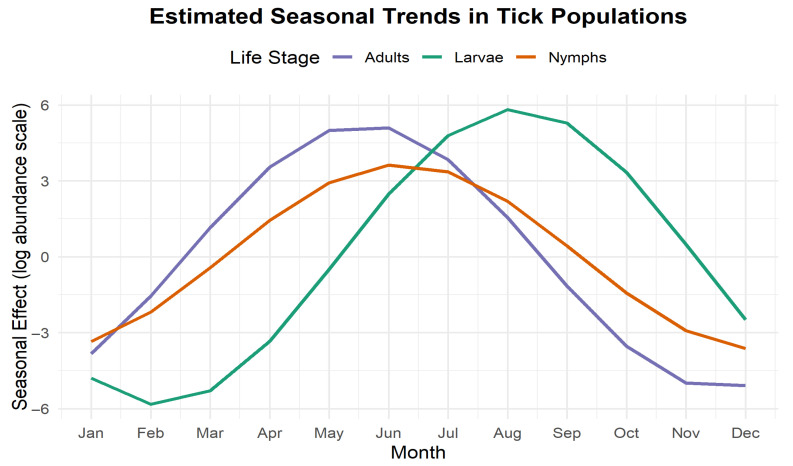
Model-estimated seasonal patterns for larvae (green), nymphs (orange), and adults (purple) derived from the NB model. Each life stage exhibits a distinct seasonal peak, reflecting biological progression through the tick life cycle.

Seasonal effects for life stage j were calculated using the posterior mean estimates of the sinusoidal coefficients as follows:

$$Season\;effect_{j}(M)=\beta_{sin,j}sin\left(\frac{2\pi \cdot M}{12}\right)+\beta_{cos,j}cos\left(\frac{2\pi \cdot M}{12}\right),$$

where $\beta_{sin,j}$ and $\beta_{cos,j}$ are the estimated sinusoidal coefficient for stage j, and M is the numeric month (with 1 denoting January and 12 denoting December). The resulting effects are illustrated in Figure 6. The figure presents model-estimated seasonal trends in tick populations across different life stages to describe the seasonal effect on a logarithmic scale. This approach captures the predictable, recurring fluctuations in tick activity over the course of a calendar year.

Adult tick activity, represented by the purple line, begins to increase in late winter, rising sharply through the spring months. Peak abundance occurs between May and June, after which there is a gradual decline throughout the summer and fall, reaching its lowest levels from November through January. This pattern suggests that adult ticks are most active in late spring and early summer.

Nymphal ticks, shown in orange, exhibit a slightly delayed but similar trend compared to adults. Their activity begins to increase in early spring, peaking in June and remaining relatively high through July. Following this peak, nymph abundance declines through the late summer and fall, reaching its minimum in the winter months. Given their role as primary vectors of tick-borne pathogens, this mid-summer peak in nymph activity is particularly relevant for public health interventions.

Larvae, represented by the green line, show a markedly different seasonal pattern. Their abundance remains very low through the winter and early spring and then begins to increase steeply in late spring. The peak in larval activity occurs in August, later than both adults and nymphs. After this peak, the larval population declines steadily through the fall and reaches its nadir in the winter. This temporal lag in peak abundance relative to the other stages reflects the developmental progression of ticks through their life cycle.

Overall, the sinusoidal model effectively captures the distinct but overlapping periods of peak activity for each life stage, reflecting the biological timing of tick development and host-seeking behaviour. These seasonal dynamics are crucial for understanding exposure risk patterns and optimizing the timing of tick control strategies. This temporal ordering of peak activity across life stages is also clearly reflected in Figure S1 (Supplementary Materials), providing empirical support for the biological progression and lagged dependencies incorporated in the model.

**Figure 7:**
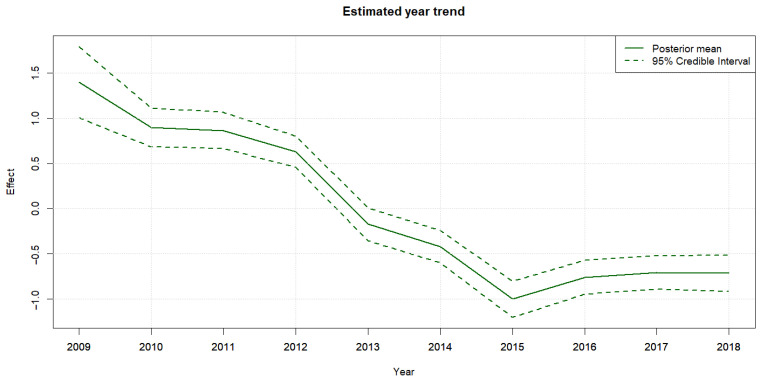
Estimated shared year effect (2009–2018) from the NB model. Solid line represents posterior mean; dashed lines show 95% credible intervals. A sustained decline in adjusted tick abundance is observed after 2013.

The estimated year effect from the negative binomial (NB) model reveals a clear temporal trend in tick abundance from 2009 to 2018. As illustrated in Figure 7, the posterior mean estimates (solid green line) show a progressive decline in the year effect over the study period, with 95% credible intervals (dashed lines) indicating the associated uncertainty.

From 2009 to 2012, the year effects are consistently positive, suggesting that tick abundance, after accounting for covariates such as habitat type, seasonal effects, and spatial structure, was relatively elevated during this early phase. Beginning in 2013, there is a noticeable and sustained decrease in the year effect, which becomes distinctly negative by 2015. During this period, credible intervals in this period do not cross zero, indicating a statistically meaningful downward trend.

Between 2015 and 2018, the year effect appears to stabilize at lower levels, with only modest increases observed. Nonetheless, the posterior means remain below zero, indicating that adjusted tick abundance was consistently suppressed relative to the earlier years. The narrowing of credible intervals in later years further reflects the increasing certainty in these estimates.

## 4. Discussion

This study examined the spatio-temporal abundance patterns of *Amblyomma americanum* (lone star tick) life stages in Virginia using negative binomial (NB) and zero-inflated negative binomial (ZINB) models. While the ZINB model yielded marginally lower information criteria, the NB model was preferred due to its biological interpretability and stability. In particular, the NB formulation preserved positive inter-stage dependencies that are consistent with the tick life cycle, where progression from larvae to nymphs and adults depends on the survival and abundance of preceding stages. This aligns with recommendations that model selection in ecological studies should prioritize biological realism alongside statistical performance [29]. In contrast, the ZINB model exhibited instability associated with excessive zero inflation, a known limitation in ecological count data that can distort inferred relationships [30].

The pronounced seasonal patterns observed across tick life stages are consistent with established ixodid tick phenology. Peaks in abundance during warmer months reflect the strong influence of temperature and humidity on tick development and host-seeking behaviour [31]. Habitat-specific effects further supports this interpretation, with higher abundance in wooded habitats compared to grassy areas, likely due to favourable microclimatic conditions that reduce desiccation and enhance survival. Similar habitat associations have been reported in prior studies, emphasizing the role of local environmental heterogeneity in shaping tick populations [13,32].

The positive lagged effects linking successive life stages provide additional ecological validation of the modelling framework. These findings are consistent with life cycle models in which population sizes at later stages depend on the survival and abundance of earlier stages [33]. Together, the seasonal, habitat and lagged effects highlight the value of a multivariate approach that explicitly accounts for biological dependencies across tick life stages, which cannot be achieved via univariate model approaches.

The shared year effect estimated by the NB model revealed a sustained decline in adjusted tick abundance beginning around 2013 and persisting through 2018. Such multi-year declines have been documented in other vector systems and are often attributed to broader ecological pressures rather than short-term stochastic variation [34]. Periods of prolonged drought or extreme heat can suppress tick populations across multiple life stages, potentially producing sharp declines such as that observed between 2013 and 2015. One plausible explanation is interannual climatic variability, as temperature, humidity, and precipitation strongly regulate tick survival, development, and questing activity [3,31,35]. Land-use change and habitat fragmentation may also contribute to longer-term trends by altering microclimate and host community structure, thereby influencing tick persistence and reproduction [36].

In addition, host dynamics are likely to play a role. White-tailed deer are key reproductive hosts for adult *A. americanum*, while small mammals support immature stages; fluctuations in these host populations can propagate through the life cycle and generate multi-year changes in abundance [37,38,39]. Although climate and host data were not explicitly incorporated in the present analysis, the observed temporal patterns are consistent with these well-established ecological mechanisms and warrant further investigation.

The seasonal patterns identified in this analysis have direct relevance for public health surveillance and intervention strategies in Virginia. The period of elevated tick abundance between May and August coincides with peak seasonal incidence of tick-borne diseases associated with *A. americanum*, including human monocytic ehrlichiosis and Heartland virus diseases, which are most frequently reported during the late spring and summer in the Mid-Atlantic and southeastern United States [40]. This temporal alignment suggests that targeted interventions during this window may be particularly effective in reducing human exposure risk.

From a public health perspective, these findings support prioritizing enhanced tick surveillance, public awareness campaigns, and targeted control measures during early to mid-summer, particularly in wooded and edge habitats where tick abundance is highest. Control strategies such as habitat management, targeted acaricide application in high-risk recreational areas, and deer-focused interventions prior to or during peak seasonal activity [41]. In addition, intensified monitoring of tick populations and associated pathogens during this period can improve early detection of risk, including emerging and re-emerging pathogens and inform timely public health responses.

### Limitations and Further Research

Several limitations should be acknowledged. The temporal scope of the dataset ends in 2018 and therefore does not capture more recent trends, including potential disruptions to surveillance during the COVID-19 pandemic [7]. The analysis was restricted to a subset of well-sampled locations, which may limit generalizability across all Virginia counties, a common challenge in tick surveillance studies [41]. Furthermore, the absence of egg-stage data and direct measurements of climate and host abundance constrained a more comprehensive evaluation of underlying drivers.

Future research should extend the temporal coverage, incorporate additional life stages and integrate climate and host data within joint spatio-temporal modelling frameworks. Such extensions would enable more explicit testing of ecological hypotheses and further enhance the utility of statistical models for informing targeted public health interventions. A model extension to capture both the tick and disease dynamics would most exhaustively be more informative from a public health point of view.

## 5. Conclusions

The NB model offers a robust framework for understanding lone star tick dynamics, supported by the ecological and epidemiological literature. The findings advocate for seasonal, habitat type-focused interventions in Virginia, particularly in wooded areas during peak activity periods, to address the growing public health threat of tick-borne diseases. These insights provide a foundation for adaptive management and further research to refine control strategies in response to ongoing environmental changes. The models can be applied to tick and tick-borne disease data beyond the USA, including regions such as Africa, where the emphasis is on animal diseases that affect livestock production and hinder food security efforts.

## Figures and Tables

**Table 1:** Summary diagnostics statistics for tick life stage count data.

Metric	Larvae	Nymphs	Adults
Mean	56.10	5.90	3.85
Variance	75,891.00	766.00	182.00
Variance-to-Mean (VMR)	1352.00	130.00	47.10
Proportion Zero	0.83	0.64	0.70
Expected Zero (Poisson)	0.00	0.00	0.03

**Table 2:** Model comparison based on Deviance Information Criterion (DIC) and Watanabe–Akaike Information Criterion (WAIC).

Model	Count Distribution	DIC	WAIC
1	Negative Binomial	11,251.57	11,280.74
2	Zero-Inflated NB	11,242.28	11,271.14

**Table 3:** Posterior summaries and model diagnostics under three prior sets for multivariate NB-INLA model.

Parameter	Set 1: Mean (SD) [95% CI]	Set 2: Mean (SD) [95% CI]	Set 3: Mean (SD) [95% CI]
Fixed Effects $\left(\beta_{j}\right)$			
$\beta_{sin,\;L}$	−5.285 (0.229) [−5.736, −4.837]	−5.288 (0.231) [−5.745, −4.837]	−5.290 (0.232) [−5.748, −4.836]
$\beta_{sin,N}$	−0.430 (0.096) [−0.617, −0.242]	−0.429 (0.096) [−0.616, −0.242]	−0.430 (0.096) [−0.618, −0.242]
$\beta_{sin,A}$	1.154 (0.106) [0.945, 1.362]	1.152 (0.106) [0.943, 1.360]	1.153 (0.107) [0.944, 1.363]
$\beta_{cos,L}$	−2.483 (0.205) [−2.885, −2.082]	−2.488 (0.205) [−2.889, −2.086]	−2.486 (0.205) [−2.889, −2.084]
$\beta_{{cos}_{N}}$	−3.624 (0.133) [−3.887, −3.363]	−3.621 (0.137) [−3.892, −3.355]	−3.622 (0.135) [−3.889, −3.358]
$\beta_{{cos}_{A}}$	−5.093 (0.197) [−5.481, −4.707]	−5.088 (0.200) [−5.480, −4.697]	−5.091 (0.200) [−5.484, −4.701]
$\beta_{Grass}$	−1.298 (0.094) [−1.482, −1.114]	−1.298 (0.094) [−1.481, −1.114]	−1.299 (0.094) [−1.483, −1.115]
$\beta_{Woods}\;$	0.496 (0.090) [0.320, 0.673]	0.496 (0.090) [0.320, 0.672]	0.496 (0.090) [0.319, 0.672]
$\beta_{\log\left(L_{t-1}+1\right)}$	0.212 (0.034) [0.145, 0.280]	0.212 (0.035) [0.144, 0.280]	0.212 (0.035) [0.144, 0.280]
$\beta_{\log\left(N_{t-1}+1\right)}$	0.107 (0.045) [0.019, 0.194]	0.107 (0.044) [0.020, 0.194]	0.107 (0.045) [0.019, 0.194]
Overdispersion Parameters $\left(\phi_{j}\right)$			
$\phi_{L}$	0.077 (0.005) [0.068, 0.087]	0.077 (0.006) [0.065, 0.090]	0.077 (0.005) [0.068, 0.088]
$\phi_{N}$	0.467 (0.026) [0.417, 0.520]	0.467 (0.029) [0.413, 0.526]	0.467 (0.030) [0.412, 0.528]
$\phi_{A}$	1.061 (0.076) [0.919, 1.219]	1.064 (0.099) [0.884, 1.273]	1.062 (0.089) [0.899, 1.248]
Precision of Year Effect $\left(\tau_{year}\right)$			
$\tau_{year}$	6.912 (0.948) [5.198, 8.924]	20.284 (40.867) [0.835, 110.488]	6.963 (1.519) [4.423, 10.375]
Spatial Precision $\left({\tau_{loc3d}}_{j}\right)$			
${\tau_{loc3d}}_{L}$	1.601 (0.188) [1.262, 1.999]	1.842 (1.208) [0.453, 4.985]	1.617 (0.311) [1.088, 2.306]
${\tau_{loc3d}}_{N}$	1.749 (0.240) [1.319, 2.262]	1.775 (0.509) [0.970, 2.954]	1.763 (0.259) [1.304, 2.320]
${\tau_{loc3d}}_{A}$	0.263 (0.031) [0.207, 0.330]	0.264 (0.046) [0.185, 0.364]	0.270 (0.056) [0.175, 0.396]
Stage Correlations $\left(\rho_{jk}\right)$			
$\rho_{L:N}$	0.667 (0.036) [0.591, 0.735]	0.670 (0.056) [0.550, 0.769]	0.663 (0.055) [0.545, 0.760]
$\rho_{L:A}$	0.456 (0.047) [0.362, 0.546]	0.465 (0.129) [0.189, 0.692]	0.457 (0.067) [0.322, 0.585]
$\rho_{N:A}$	0.787 (0.028) [0.727, 0.837]	0.781 (0.054) [0.657, 0.868]	0.782 (0.041) [0.692, 0.853]
Model Fit			
DIC	11,249.96	11,247.48	11,250.83
WAIC	11,278.82	11,275.31	11,279.88

## Data Availability

The dataset used in the current study can be made available upon reasonable request.

## Supplementary Materials

The following supporting information can be downloaded at: https://www.mdpi.com/article/10.3390/ijerph23050660/s1. File S1: Data tables: Table S1. Geographic and site-specific information for all tick sampling sites included in the study. The table lists site identifiers, habitat types, geographic coordinates (latitude and longitude), and corresponding municipalities in southeastern Virginia, Table S2. Number of sampling visits conducted across all locations and habitat types (Edge, Grass, and Woods) for *Amblyomma americanum* tick data collected in southeastern Virginia (2009–2018), Table S3. Number of sampling events across the six retained locations (BB, BW, KP, LA, and OD) and three habitat types (Edge, Grass, and Woods) used in the final multivariate modelling dataset (2009–2018); File S2: Plots: Figure S1. Monthly time series of *Amblyomma americanum* tick life stage counts (larvae, nymphs, and adults) from May 2009 to December 2018, illustrating seasonal patterns, variability, and temporal dependencies across life stages, Figure S2. Map of Virginia showing the geographic distribution of sampled countries, with study locations highligted and labelled, Figure S3. Zoomed-in map of southeastern Virgnia showing detailed spatial distribution of sampled countries and tick collection sites, Figure S4. Percentage distribution of zero tick counts for *Amblyomma americanum* across habitat types (Edge, Grass, and Woods) from 2009 to 2018, Figure S5. Monthly percentage distribution of zero tick counts for *Amblyomma americanum*, illustrating seasonal variation in absence of tick activity (2009–2018), Figure S6. Joint percentage distribution of zero tick counts for *Amblyomma americanum* across habitat types and months, highlighting combined environmental and seasonal effects (2009–2018).; File S3: Technical model specification; File S4: Model Results: Diagnostic Plots: Figure S7. Posterior predictive checks comparing observed mean tick counts (solid grey) with fitted mean counts from the Negative Binomial (NB; solid black) and Zero-Inflated Negative Binomial (ZINB; dashed black) models for adults, larvae, and nymphs over time. The plots assess how well each model reproduces the overall temporal patterns and seasonal peaks observed in the data, Figure S8. Scatter plots of Pearson-type residuals against fitted (posterior mean) values for the NB and ZINB models, shown separately for adults, larvae, and nymphs. These plots are used to assess systematic patterns, model bias, and potential overfitting, Figure S9. Probability Integral Transform (PIT) histograms for the ZINB model for adults, larvae, and nymphs. Under a well-calibrated model, PIT values are expected to be approximately uniformly distributed between 0 and 1 (indicated by the dashed horizontal line).
