# Supplementary material for: Bayesian Multivariate Modelling of Lone Star (Amblyomma americanum) Tick Life Stage Abundance and Temporal Trends to Inform Public Health Risk in Virginia
Source: Int J Environ Res Public Health. 2026 May 15;23(5):660. doi: 10.3390/ijerph23050660 (PMC13207010; doi:10.3390/ijerph23050660)
Supplement: Supplementary file 1 [file ijerph-23-00660-s001.zip › ijerph-3904377-supplementary.pdf]

File S1: Data tables

Table S1: Geographic and site-specific information for all tick sampling sites included in the study. The table lists site identifiers, habitat types, geographic coordinates (latitude and longitude), and corresponding municipalities in southeastern Virginia.

ID	Site ID	Transect	Site Name	Habitat	Latitude	Longitude	Municipality
1	JC1	1	Transect 1	Grass	36.8	-76.4	Chesapeake City
2	JC2	2	Transect 2	Grass	36.8	-76.4	Chesapeake City
3	JC5	5	Other	Edge	36.8	-76.4	Chesapeake City
4	ST1	1	Grass	Grass	36.6	-76.3	Chesapeake City
5	LA1	1	CDC	Edge	37.1	-76.4	Hampton City
6	LA2	2	Munitions	Woods	37.1	-76.4	Hampton City
7	LA3	3	Popular Grass	Grass	37.1	-76.4	Hampton City
8	LA4	4	Popular Woods	Woods	37.1	-76.4	Hampton City
11	BW1	1	Burned upland	Woods	36.8	-76.9	Isle of Wight
12	BW2	2	Burned bottomland	Woods	36.8	-76.9	Isle of Wight
13	BW3	3	No burn upland	Woods	36.8	-76.9	Isle of Wight
14	BW4	4	No burn bottomland	Woods	36.8	-76.9	Isle of Wight
15	WS1	1	Trails	Woods	36.9	-76.3	Norfolk City
16	KP1	1	Grass	Grass	37.2	-76	Northampton City
17	KP2	2	Sand trail	Woods	37.2	-76	Northampton City
18	KP3	3	Beach	Edge	37.2	-76	Northampton City
21	PC1	1	Grass	Grass	36.8	-76.3	Portsmouth City
22	PC2	2	Woods	Woods	36.8	-76.3	Portsmouth City
23	BB1	1	Kuralt Trail	Edge	36.7	-75.9	Virginia Beach City
24	BB2	2	Grass	Grass	36.7	-75.9	Virginia Beach City
25	BB3	3	Dune Trail	Edge	36.7	-75.9	Virginia Beach City
26	OD1	1	Child Development Center	Edge	36.8	-76	Virginia Beach City
27	OD2	2	Back Gate	Woods	36.8	-76	Virginia Beach City
28	OD3	3	Beach/Dune	Grass	36.8	-76	Virginia Beach City
29	OD4	4	Windmills	Edge	36.8	-76	Virginia Beach City
30	CA1	1	Grass	Grass	37.3	-76.6	York County
31	CA2	2	Woods	Woods	37.3	-76.6	York County
32	CA3	3	River	Woods	37.3	-76.6	York County
33	CA4	4	C@C	Edge	37.3	-76.6	York County
34	NN1	1	R4	Edge	37.2	-76.5	York County

Table S2: Number of sampling visits conducted across all locations and habitat types (Edge, Grass, and Woods) for *Amblyomma americanum* tick data collected in southeastern Virginia (2009 - 2018).

LOCATION	Edge	Grass	Woods	Total
BB	90	90	0	**180**
BW	0	69	74	**143**
CA	89	96	92	**277**
HC	0	0	96	**96**
JC	2	62	0	**64**
KP	69	68	69	**206**
LA	73	74	75	**222**
NN	0	0	15	**15**
OD	98	87	94	**279**
PC	0	24	79	**103**
ST	94	0	0	**94**
WS	0	0	64	**64**
**Total**	**515**	**570**	**658**	**1743**

Table S3: Number of sampling events across the six retained locations (BB, BW, KP, LA, and OD) and three habitat types (Edge, Grass, and Woods) used in the final multivariate modelling dataset (2009 - 2018).

LOCATION	Edge	Grass	Woods	Total
BB	90	90	0	180
BW	0	69	74	143
CA	89	96	92	277
KP	69	68	69	206
LA	73	74	75	222
OD	98	87	94	279
**Total**	**419**	**484**	**404**	**1307**

Table S3 summarizes the number of sampling events across the six retained locations and three habitat types (Edge, Grass, and Woods). A value of zero in this table indicates that no sampling visits were conducted in that habitat type at the corresponding location, and therefore no data were collected. These structural gaps are important to consider, as they reflect limitations in habitat coverage rather than zero tick abundance. For example, wooded habitats were not sampled at BB, and edge habitats were not sampled at BW.

File S2: Plots

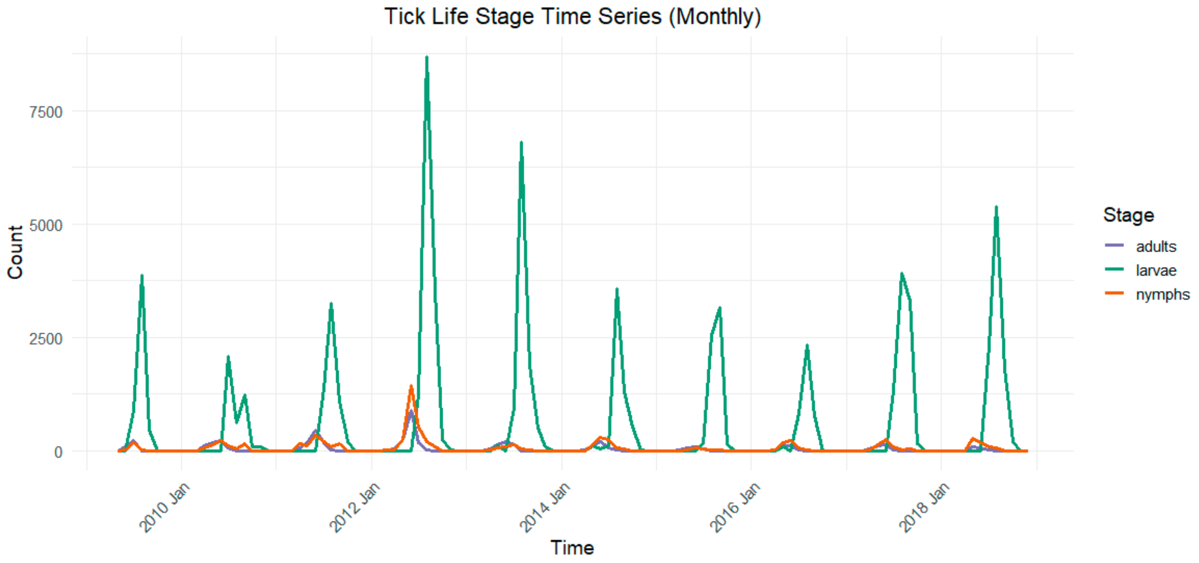

Figure S1. Monthly time series of *Amblyomma americanum* tick life stage counts (larvae, nymphs, and adults) from May 2009 to December 2018, illustrating seasonal patterns, variability, and temporal dependencies across life stages.

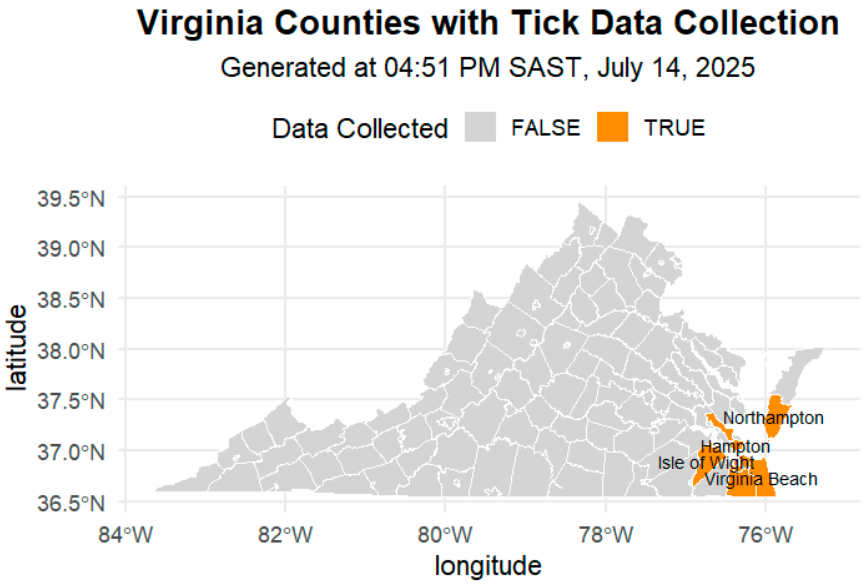

Figure S2. Map of Virginia showing the geographic distribution of sampled countries, with study locations highligted and labelled.

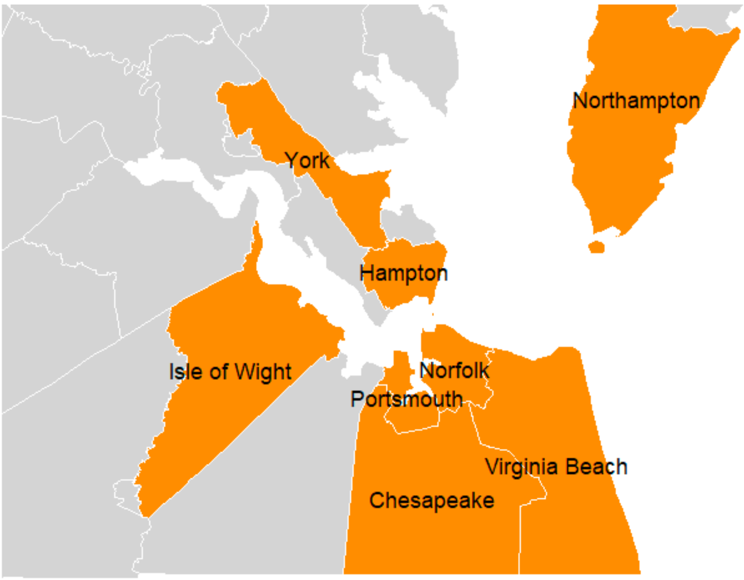

Figure S3. Zoomed-in map of southeastern Virgnia showing detailed spatial distribution of sampled countries and tick collection sites.

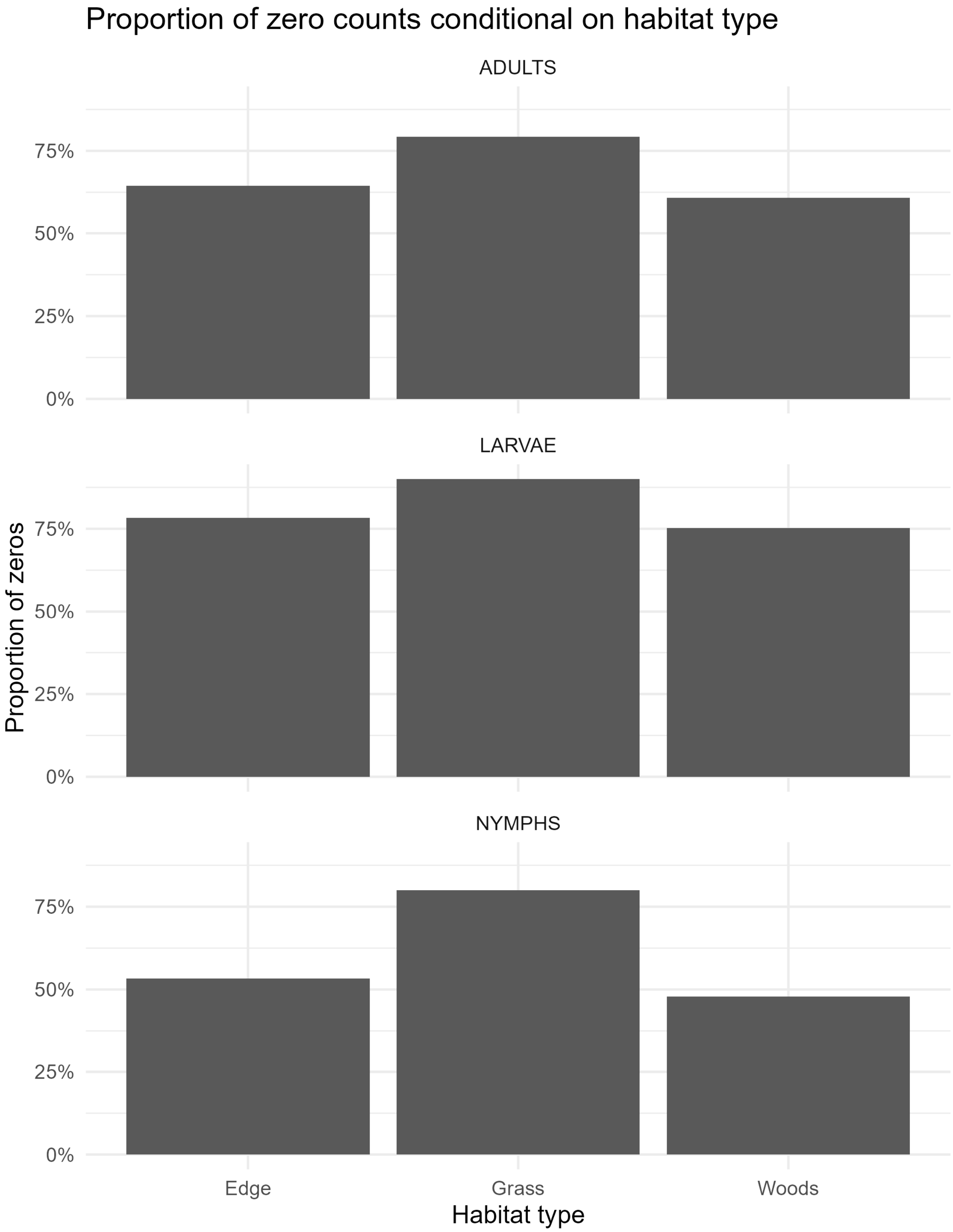

Figure S4. Percentage distribution of zero tick counts for *Amblyomma americanum* across habitat types (Edge, Grass, and Woods) from 2009 to 2018.

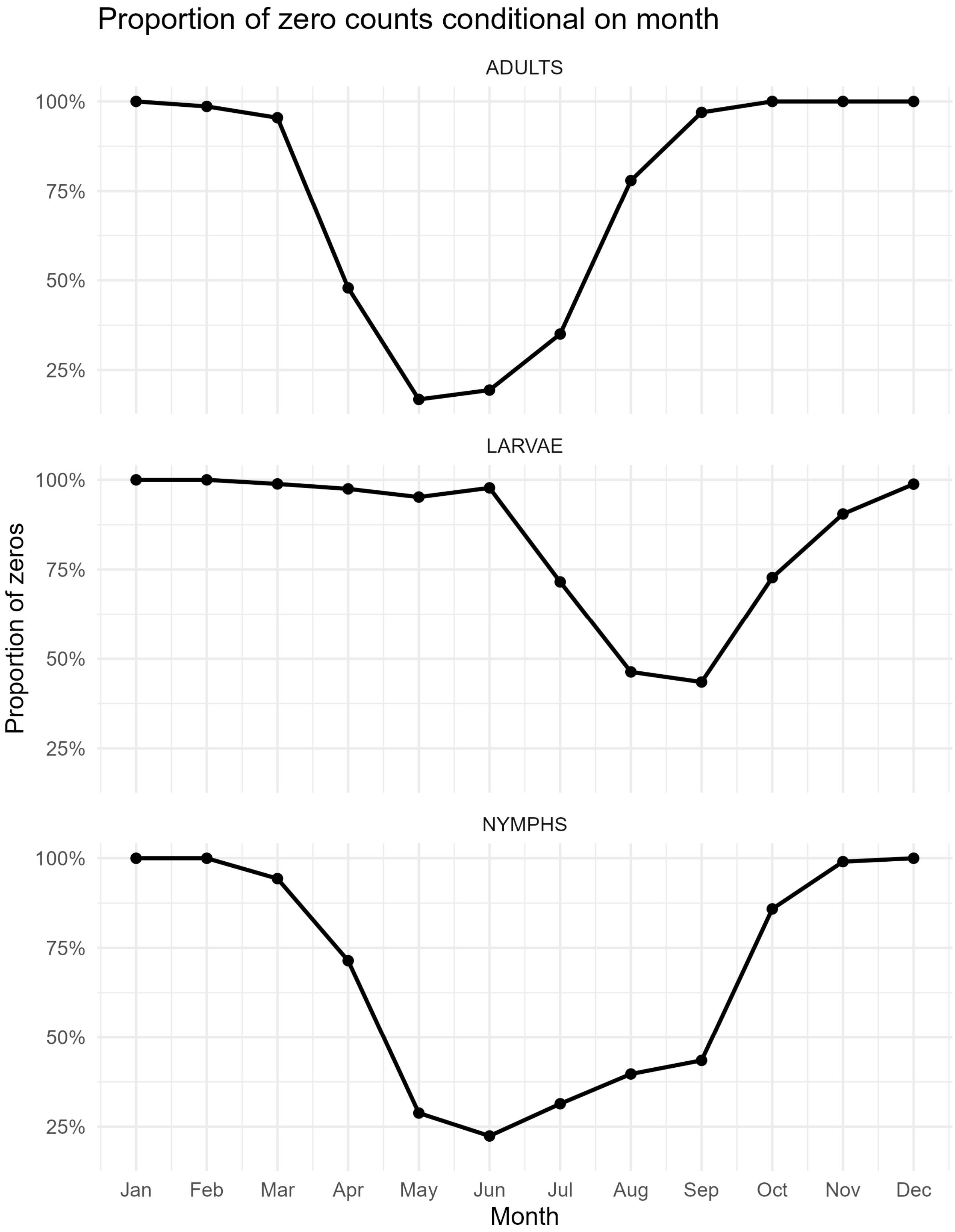

Figure S5. Monthly percentage distribution of zero tick counts for *Amblyomma americanum*, illustrating seasonal variation in absence of tick activity (2009 - 2018).

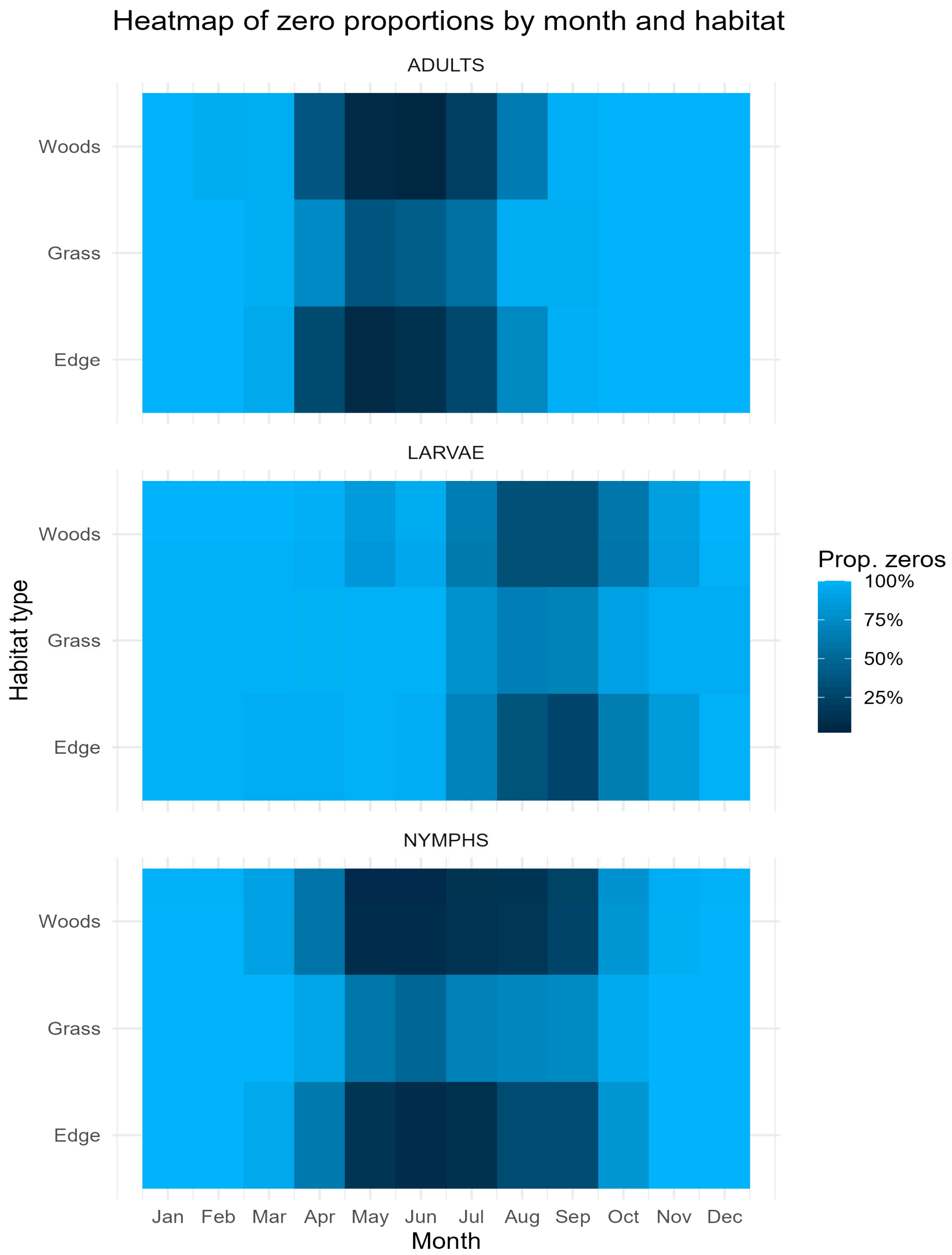

Figure S6. Joint percentage distribution of zero tick counts for *Amblyomma americanum* across habitat types and months, highlighting combined environmental and seasonal effects (2009 - 2018).

File S3: Technical model specification

To understand the temporal and spatial dynamics of Lone Star Tick *A. americanum* abundance across life stages, we model the multivariate count data using the negative binomial (NB) and the zero-inflated negative binomial (ZINB). These models were selected to account for key characteristics of the observed data, including overdispersion and an excess of zeros. The NB model allows for over-dispersed count data by introducing a dispersion parameter, while the ZINB model extends the NB by further accounting for structural zeros due to ecological or sampling processes that lead to true absence.

For each tick life stage j, at location i, and time t, let $Y_{j,it}$ denote the observed count. Under the NB model, we assume $Y_{j,it} \sim \text{NB}(\lambda_{j,it}, \phi_j)$, where $\lambda_{j,it}$ is the expected count for stage j at location i, and at time t, and ϕ_j is the stage-specific dispersion parameter. To account for the pronounced zero-inflation evident in the exploratory data analysis, we consider a ZINB model, where $Y_{j,ikt} \sim \text{ZINB}(\lambda_{j,it}, \phi_j, \pi_{j,it})$. Here, $\pi_{j,it}$ represents the probability of a structural zero; that is, a count of zero not generated by the NB process, and $\lambda_{j,ikt}$ and ϕ_j retain their interpretations as in the NB model. In both models, the log-expected count $\lambda_{j,ikt}$ is linked to covariates using the log-link function as follows:

$$\log(\lambda_{j,ikt}) = S'_{j,ikt}\beta_j + v_t + b_k,$$

where $S'_{j,ikt}$ is a vector of covariates for stage j, location i, and time t, β_j are the associated regression coefficients for the fixed effects.

Covariates include habitat type (with edge habitat type as reference), sinusoidal terms to capture the seasonality in the life stage (Figure S1), and lagged effects of preceding stages to reflect biological transition dynamics: one- month lags of larvae are included to assess effects on nymphs, and similarly only one-month lagged effects of nymphs to assess effects on adults. We did not include any lagged effects of adults on eggs laid since this data was not collected. The omission of an intercept ensured that all levels of the categorical covariates were explicitly parameterized relative to the reference categories.

To capture temporal trends either shared or common across all tick life stages, a first-order random walk $v_t \sim \text{N}(v_{t-1}, \tau_{year}^{-1})$ is specified. The term v_t represents a random walk effect for time (yearly trend, shared or common across stages) specified as $v_t \sim \text{N}(v_{t-1}, \tau_{year}^{-1})$, where τ_{year} is the precision with a log-gamma hyperprior, such that $\tau_{year} \sim \text{LogGamma}(1{,}0.00005)$. The hyperprior is set as a more informative prior $\tau_{year} \sim \text{LogGamma}(2{,}0.01)$ and as a less informative prior $\tau_{year} \sim \text{LogGamma}(1{,}0.00001)$ to assess prior sensitivity. The term b_k denotes the spatial-stage random effect for location-stage combination k, where k ranges over $3 \times n_{locs}$, where $n_{locs} = 6$ for the selected locations in the dataset, and is modelled as $b_k \sim \text{N}(0, \Sigma_{loc3d})$, where Σ_{loc3d} is a 3×3 covariance matrix with precision $\tau_{loc3d} \sim \text{LogGamma}(1{,}0.00005)$, and as $\tau_{loc3d} \sim \text{LogGamma}(2{,}0.01)$ and as $\tau_{loc3d} \sim \text{LogGamma}(1{,}0.00001)$ for the sensitivity analysis. The

zero-inflation probability $\pi_{j,it}$ is modelled on the logit scale with normal hyperprior, $\text{logit}(\pi_{j,it}) \sim N(0, \sigma_{zi}^2)$, where $\sigma_{zi}^2 = 0.1$ by default, and with sensitivity settings $\sigma_{zi}^2 = 0.5$ for more informative priors and $\sigma_{zi}^2 = 0.05$ for less informative priors. The stage-specific dispersion parameter $\phi_j \sim \text{Gamma}(a_\phi, b_\phi)$, with a and b typically set with weak parameters, $a = 1$ and $b = 0.001$ and finally, the fixed effect parameter coefficients is assigned weakly informative Gaussian priors, $\beta_j \sim \text{N}(0, 10^6)$. These priors were selected to ensure that our conclusions would not hinge on arbitrary choices, and followed best practices for Bayesian model checking.

File S4: Model Results

Diagnostic Plots

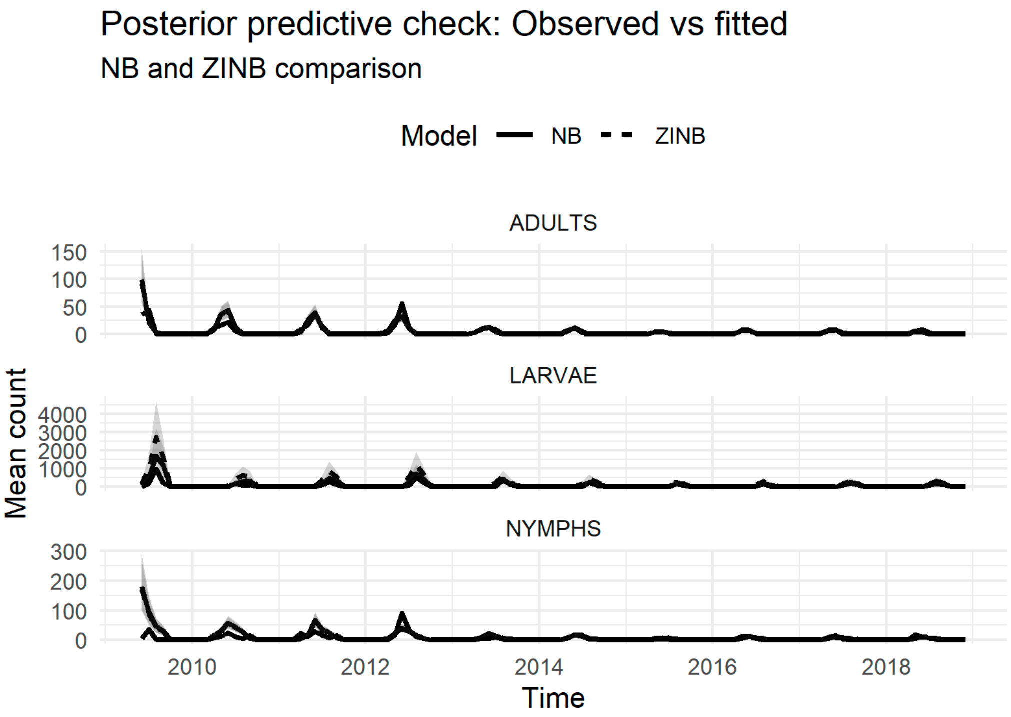

Figure S7. Posterior predictive checks comparing observed mean tick counts (solid grey) with fitted mean counts from the Negative Binomial (NB; solid black) and Zero-Inflated Negative Binomial (ZINB; dashed black) models for adults, larvae, and nymphs over time. The plots assess how well each model reproduces the overall temporal patterns and seasonal peaks observed in the data.

Interpretation (plain language)

- Both models capture the main seasonal patterns in tick abundance, including the timing of peaks and low-activity periods.

- The NB model closely tracks the observed data without producing exaggerated peaks.
- The ZINB model tends to slightly overestimate some peaks, particularly for larvae, suggesting it may be fitting noise rather than true signal.
- Overall, this comparison shows that the simpler NB model reproduces the observed patterns just as well as, and sometimes more realistically than, the more complex ZINB model.

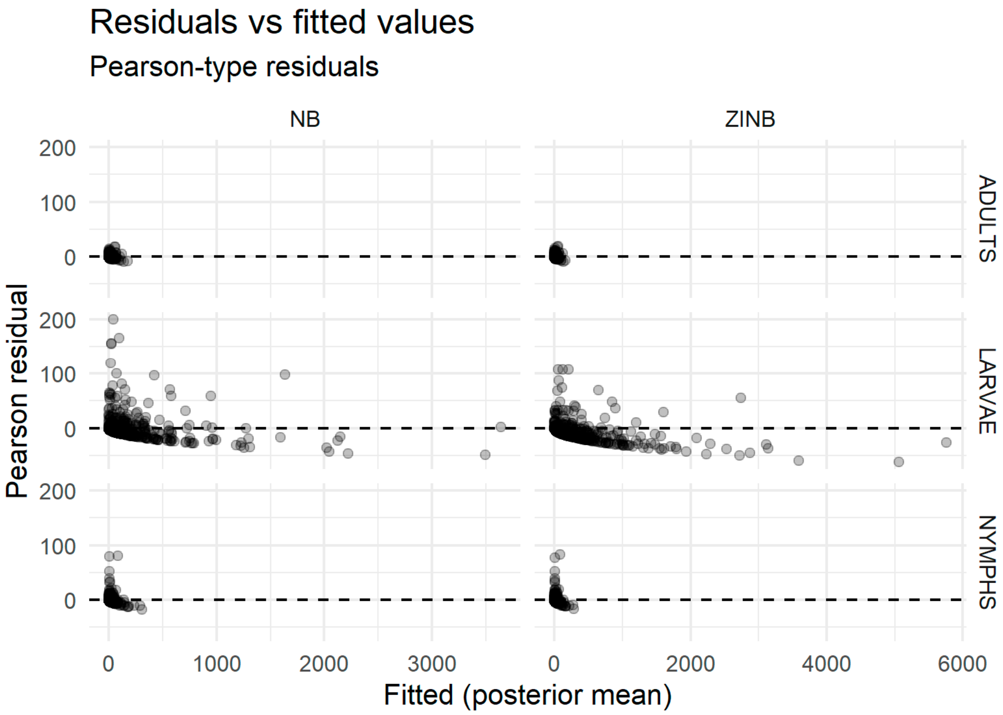

Figure S8. Scatter plots of Pearson-type residuals against fitted (posterior mean) values for the NB and ZINB models, shown separately for adults, larvae, and nymphs. These plots are used to assess systematic patterns, model bias, and potential overfitting.

Interpretation (plain language)

- For the NB model, residuals are generally centered around zero with no strong patterns, indicating a good overall fit.
- The presence of extreme residuals under the ZINB model indicates that it may be overfitting rare high-count observations.
- These diagnostics suggest that the NB model provides a more stable and consistent representation of tick counts across their full range.

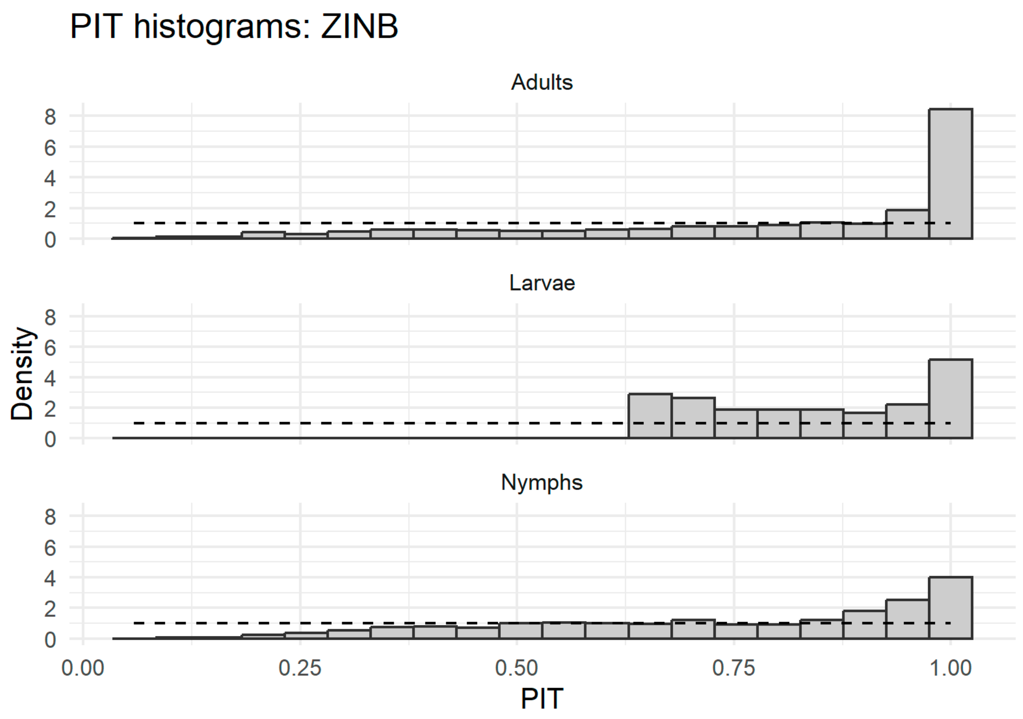

Figure S9. Probability Integral Transform (PIT) histograms for the ZINB model for adults, larvae, and nymphs. Under a well-calibrated model, PIT values are expected to be approximately uniformly distributed between 0 and 1 (indicated by the dashed horizontal line).

Interpretation (plain language)

- Instead of being evenly distributed, PIT values are strongly concentrated near 1 for all life stages.
- This pattern indicates that the ZINB model systematically underestimates observed counts, particularly during high-abundance periods.
- The accumulation of PIT values near the upper boundary suggests model miscalibration rather than random variation.
- These results provide further evidence that the additional zero-inflation component does not improve model performance and may distort inference.

ZINB model Results

```
Time used:
    Pre = 0.767, Running = 77.2, Post = 0.357, Total = 78.3
Fixed effects:
                      mean    sd 0.025quant 0.5quant 0.975quant   mode kld
sin_l               -4.760 0.186     -5.124   -4.759     -4.396 -4.759   0
```

```
sin_n               -0.426 0.095     -0.613   -0.426     -0.240 -0.426   0
sin_a                1.166 0.105      0.959    1.166      1.372  1.166   0
cos_l               -2.512 0.168     -2.841   -2.512     -2.184 -2.512   0
cos_n               -3.596 0.131     -3.853   -3.596     -3.340 -3.596   0
cos_a               -5.081 0.191     -5.456   -5.081     -4.706 -5.081   0
HABITAT_all_Grass   -1.256 0.091     -1.435   -1.256     -1.077 -1.256   0
HABITAT_all_Woods    0.475 0.087      0.305    0.475      0.646  0.475   0
larvae_lag1_aligned  0.213 0.034      0.145    0.213      0.280  0.213   0
nymphs_lag1_aligned  0.120 0.044      0.033    0.120      0.206  0.120   0

Random effects:
  Name       Model
    YEAR_all RW1 model
   loc3d IID3D model

Model hyperparameters:
                                                              mean    sd 0.025quant 0.5quant 0.975quant   mode
size for nbinomial_1 zero-inflated observations              0.345 0.114      0.185    0.324      0.629  0.283
zero-probability parameter for zero-inflated nbinomial_1     0.605 0.084      0.437    0.607      0.763  0.607
size for nbinomial_1 zero-inflated observations[2]           0.537 0.219      0.241    0.494      1.088  0.418
zero-probability parameter for zero-inflated nbinomial_1[2]  0.010 0.004      0.004    0.009      0.019  0.007
size for nbinomial_1 zero-inflated observations[3]           1.118 0.313      0.622    1.078      1.844  1.004
zero-probability parameter for zero-inflated nbinomial_1[3]  0.012 0.004      0.006    0.011      0.023  0.010
Precision for YEAR_all                                       7.954 3.619      3.006    7.261     16.933  6.030
Precision for loc3d (component 1)                            1.498 0.455      0.799    1.433      2.573  1.311
Precision for loc3d (component 2)                            1.941 0.872      0.762    1.771      4.117  1.474
Precision for loc3d (component 3)                            0.274 0.108      0.123    0.255      0.540  0.220
Rho1:2 for loc3d                                            -0.189 0.137     -0.452   -0.191      0.083 -0.189
Rho1:3 for loc3d                                            -0.605 0.140     -0.823   -0.624     -0.278 -0.665
Rho2:3 for loc3d                                             0.762 0.054      0.640    0.767      0.852  0.779

Deviance Information Criterion (DIC) ...............: 11242.28
Deviance Information Criterion (DIC, saturated) ....: 2270.24
Effective number of parameters .....................: 32.87

Watanabe-Akaike information criterion (WAIC) ...: 11271.14
Effective number of parameters .................: 54.31
```
